# Activation of sirtuin 3 and maintenance of mitochondrial homeostasis by artemisinin protect against diclofenac-induced kidney injury in rats

**DOI:** 10.1007/s00210-024-03620-8

**Published:** 2024-11-23

**Authors:** Doaa Hellal, Sarah Ragab Abd El-Khalik, Heba M. Arakeep, Doaa A. Radwan, Hend S. Abo Safia, Eman A. E. Farrag

**Affiliations:** 1 Clinical Pharmacology Department, Faculty of Medicine, Mansoura University, Mansoura, 31516 Egypt; 2 Medical Biochemistry & Molecular Biology Department, Faculty of Medicine, Tanta University, Tanta, Egypt; 3 Anatomy and Embryology Department, Faculty of Medicine, Tanta University, Tanta, Egypt; 4 Pathology Department, Faculty of Medicine, Ibn Sina Medical University, Amman, Jordan; 5 Anatomy and Embryology, Public Health Department, College of Applied Medical Science, King Faisal University, Al-Ahsa, Saudi Arabia; 6 Pathology Department, Faculty of Medicine, Tanta, Egypt

**Keywords:** DIC-induced kidney injury, Artemisinin, SIRT3, Mitochondria

## Abstract

Nonsteroidal anti-inflammatory drug (NSAID)-induced kidney injury is one of the most common causes of renal failure. The exact pathogenesis of NSAID induced kidney injury is not fully known and the treatment is still challenging. Artemisinin (ART) gains more attention by its potent biological activities in addition to its antimalarial effect. In our research, we evaluated the preventive and therapeutic effects of ART in Diclofenac (DIC) induced kidney injury through its effect on mitochondria and regulation of sirtuin 3 (SIRT3). Thirty adult male Sprague Dawley rats were divided into five groups: control, ART, DIC, DIC + ART prophylactic, and DIC followed + ART therapeutic groups. At the end of the study, animals were scarified and the following parameters were evaluated: serum urea and creatinine, renal malondialdehyde (MDA), superoxide dismutase (SOD) and nitrate. SIRT3 was detected by western blotting and real-time PCR. Mitochondrial related markers (PGC-1α, Drp1, and mitochondrial ATP) were detected by immunoassay. Caspase-3 and LC3 II expression in kidney tissues were demonstrated by immune-histochemical staining. The kidney specimens were stained for H&E and PAS special stain. Electron microscopy was done to detect mitochondrial morphology. ART improved renal function test, oxidative stress, SIRT3 level, mitochondrial function, LC3 II expression and decrease caspase-3. Histopathological examination confirmed ART alleviation as determined by light or electron microscopy. ART can modulate biochemical and pathological changes in DIC-induced kidney injury and can be considered a new possible therapeutic approach for DIC-induced kidney injury through its effect on SIR3 and maintenance of mitochondrial homeostasis.

## Introduction

The kidney is highly prone organ to damage, owing to its high blood flow, and because of its complexity both anatomically and functionally (Scholz et al. 2021). Kidney injury caused by different etiology, is associated with deterioration of kidney function and may progress to chronic renal failure (Varrier et al 2015). Emerging evidence has reported that the most common reason of acute kidney injury is ischemic and toxic agents leading to acute tubular necrosis (Amini et al. 2024). Some therapeutic drugs, such as NSAIDs, are reported to induce kidney toxicity (Harirforoosh et al 2016).

NSAIDs are considered one of the most common drug used worldwide, with an expected usage of > 30 million per day (McMahon et al. 2021). DIC is the most common NSAID, prescribed for different rheumatic disorders. Actually, DIC used annually worldwide has been assessed near 940 tons (Barzan et al. 2023; Ahmed et al. 2020).

DIC as other NSAID act by inhibiting cyclo-oxygenase enzymes with subsequent inhibition of prostaglandin (PG) which resulted in renal ischemia and injury (Verhoeven et al. 2017; Abu Hamad et al. 2017). However, inhibition of COX may not be the main mechanism of DIC-induced renal injury, and the precise mechanism of DIC-induced renal injury is not fully elucidated. It has been reported that DIC leads to mitochondrial injury by generation of reactive oxygen metabolites and inhibition of the enzymatic and non-enzymatic antioxidants in the kidney tissues (Alabi et al. 2020).

The mitochondrial disruption in kidney diseases may be related to deficiency in sirtuins (SIRTs) (Juszczak et al. 2024). SIRT3, among SIRTs, can be considered a crucial mitochondrial fidelity protein. SIRT3, a potent NAD + -dependent deacetylase, is expressed as an enzymatically inactive 44-kDa protein with an N-terminal mitochondrial targeting sequence. Inactive SIRT3 is proteolytically managed to a mature 28-kDa active enzyme that regulates mitochondrial homeostasis in response to the oxidative stress. Furthermore, it can modulate different cellular processes, including ATP generation, metabolism, apoptosis and senescence by deacetylating lysine residues of mitochondrial proteins (Zhao et al. 2018; Zhang et al. 2018). Pharmacological managements that increase SIRT3, maintaining mitochondrial integrity, may distinctly limit tissue damage accelerating recovery (Morigi et al. 2018). On attempting to discover drugs able to upregulate SIRT3 and improve mitochondrial function, artemisinin (ART) was evaluated for this purpose in DIC-induced kidney injury.

Chinese professor Youyou Tu discovered ART firstly in 1972. ART and its derivatives have the most valuable effect on malaria being used in Plasmodium vivax malaria. Now the standard antimalarial treatment all over the world is ART-combination therapies. ART use is increasing because of its effectiveness and negligible side effects (Luisi 2023).

ART has a variety of other pharmacological effects beyond antimalarial, such as anti-virus, antineoplastic, anti-inflammatory, antioxidative, and immunosuppressive effects that make it valuable in treatment of different diseases as rheumatoid arthritis, multiple sclerosis and systemic lupus erythematosus (Farrag et al. 2023; Xia et al. 2020). ART is an applicant drug that exploits the benefits of drug reuse (Meng et al. 2021).

Although several studies have demonstrated the efficacy of ART for treatment of numerous other diseases, its effects on DIC-induced kidney injury has not been illustrated. Taken all together, the current study aims to explore the novel efficiency of ART administration against DIC-induced kidney injury via maintaining of mitochondrial biogenesis and dynamics through molecular mechanisms associated with regulation of SIRT3 and oxidative stress.

## Materials and methods

### Reagents and chemicals

Diclofenac sodium (Voltarene®, 50 mg coated tablets) was acquired from Novartis pharmaceutical Co. (Cairo, Egypt). Artemisinin was acquired from (Sigma-Aldrich Chemical Co., Louis, MO, USA); it was standardized to contain a minimum of 98% artemisinin. Both drugs were diluted in 0.9% saline immediately prior administration.

### Experimental animals

In the research, thirty Sprague Dawley adult male rats (weight, 200 ± 40 g) were collected from Medical Experimental Research Center (MERC, Egypt). In accommodation previous starting the study, rat chow was the rat’s nutrition and free access to water was permitted for two weeks, rats were exposed to 12 h light/dark cycle and lived at a temperature of 25 ± 3, and the humidity was of 56 ± 3%. The study was conducted according to the National Institutes of Health guide for the care and use of Laboratory animals (NIH Publications No. 85–23, revised in 1996). This study was performed in accord the Research Ethics committee of Tanta University, Faculty of Medicine (Approval number 35250/2/22).

### Animal grouping and experimental design

The rats were haphazardly divided into five groups (6 rats/group) as follows:

1. Group I (negative control group): Rats were given 0.9% sodium chloride solution (1 ml/100 g of body weight) via oral gavage for 3 days as a vehicle.
2. Group II (ART control group): Rats were given ART (75 mg/kg) by oral gavage for 3 days (Zhang et al. 2020).
3. Group III (DIC group): Rats were given DIC at dose of (40 mg/kg/day) by oral gavage for 3 days (Abiola et al. 2019).
4. Group IV (DIC + ART prophylactic group): Rats were given ART (75 mg/kg/day) 60 min before DIC daily with previous doses for 3 days.
5. Group V (DIC followed + ART therapeutic group): Rats were given DIC at dose of 40 mg/kg/day by oral gavage for 3 days followed by ART 75 mg/kg/day by oral gavage for further 3 days.

### Blood and tissue sampling

At the end of the experiment, rats were anaesthetized via intraperitoneal injection of pentobarbital (40 mg/kg). Blood samples were collected from tail vein then we let them to clot and centrifugation was done for 10 min to obtain sera at 3000 rpm and then stored until biochemical analysis at − 20 °C. Kidneys were perfused in situ with ice cold saline after being separated from all experimental rats immediately after scarification. For histopathological examination, the right kidney for each rat was fixed in 10% formalin. The left kidney was used for preparation of tissue homogenates and RNA extraction after being dried on a filter paper, weighed and cut into small pieces that were frizzed at − 80°c till.

### Preparation of tissue homogenate and mitochondrial isolation

- One part of renal tissue was homogenized in cold buffer (i.e., 50 mM potassium phosphate, pH 7.5. 1 mM EDTA). Then, centrifugation of the renal homogenates was done for 15 min at 10000 × g at 4 °C, and the resulting supernatant was stored till used for further analysis at − 80 °C.
- The other part of renal tissue was homogenized on ice in mitochondrial isolation buffer (0.01 M Tris–HCl, 0.0001 M EDTA-2Na, 0.01 M sucrose, 0.8% NaCl, pH 7.4) by a Teflon Potter homogenizer. The homogenate was centrifuged at 1500 rpm for 5 min at 4 °C. The resultant supernatant was re-centrifuged at 10,000 rpm for 15 min to get the mitochondrial fraction (Ding et al. 2013).
- Total protein content was determined in both mitochondrial fractions and renal tissue homogenates by Lowry et al.’s method (Lowry et al. 1951).

### Biochemical assays

#### Assay of renal injury-associated serum markers

Serum levels of urea and creatinine (Cr) were measured via commercial kits (Biodiagnostic Co., Cairo, Egypt) by an enzymatic-colorimetric method consistent with the manufacturer’s procedure.

#### Assay of oxidative stress-related markers

Renal tissue malondialdehyde (MDA) level and the activity of Superoxide dismutase (SOD), express lipid peroxidation and antioxidant markers respectively, were measured using enzyme-linked immunosorbent assay kits (Biodiagnostic Co., Cairo, Egypt) according to the manufacturer’s guidance. The results were expressed as nmol/mg protein and U/mg protein, respectively.

#### Assay of renal nitrites

As an indicator of nitric oxide (NO) synthesis, nitrite levels were used. It was assessed in the supernatant of the renal homogenate via NO assay kit (Biodiagnostic Co., Cairo, Egypt) following the manufacturer’s instruction with expression of the results as μmol/mg protein.

#### Immunoassay of mitochondrial related markers

An enzyme-linked immunosorbent assay was used to measure the levels of PGC-1α (MBS762203), dynamin-related protein 1 (Drp1), and mitochondrial ATP (Cat. No. K354–100) in mitochondrial fraction using commercial kits (MyBiosource, Inc., San Diego, USA; Sun Red Biological Technology, Shanghai, China; and BioVision, Mountain View, CA, USA, respectively) in line with the manufacturers’ guidance. The color intensity was observed by absorbance at 450 nm using a microplate reader (Stat Fax 2100, New York, USA).

### Quantitative estimation of SIRT3 relative mRNA gene abundance by real-time PCR

Total RNA isolation from renal tissue samples involved using Gene JET RNA Purification Kit following the kit guidance (Thermo Scientific, #K0731, USA). RNA was reverse transcribed for cDNA production by means of Revert Aid H Minus Reverse Transcriptase (Thermo Scientific, #EP0451, USA). Finally, cDNA was used as a template to identify SIRT3 relative gene expression by step one plus real time PCR system (Applied Biosystem, USA). The primers sequences were as follows: SIRT3 (NCBI GenBank Nucleotide accession # NM_001106313.2) gene, F (5′-AAGACATACGGGTGGAGCCT-3′; reverse (5′-GGACTCAGAGCAAAGGACCC-3′) with β-actin (NCBI GenBank Nucleotide accession # NM_031144.3) as the housekeeping gene with primer sequence forward: 5′-CCCGCGAGTACAACCTTCTT-3′; reverse: 5′-CGACGAGCGCAGCGATA-3′. The cycle threshold (Ct) values for the target and housekeeping genes were determined, and relative gene expression was calculated using the 2 − ∆∆Ct method (Livak and Schmittgen 2001).

### Western blotting

The levels of mitochondrial SIRT3 was detected using western blotting. The total protein was extracted. The Ready PrepTM protein extraction kit (total protein) provided by Bio-Rad Inc. (Catalog #163–2086) was employed consistent with the manufacturer’s guidance. Following protein concentration assay, an aliquot of 20 μg proteins was separated on SDS-PAGE gel and blotted onto a piece of the PVDF membrane (Amersham Biosciences). Then, the membranes were washed with Tris-buffered saline-Tween (TBST) and blocked with 3% bovine serum albumin (Sigma-Aldrich, USA) in TBST for 1 h at 4° C. Subsequent, the membranes were incubated with the primary antibodies against SIRT3 (dilution 1: 500, Abcam, Cambridge) overnight at 4° C, followed by incubation with peroxidase-conjugated secondary antibodies (Goat anti-rabbit IgG-HRP-1 mg Goat mab-Novus Biologicals) for 1 h at room temperature. Finally, target proteins on membranes were visualized by the chemiluminescent substrate (Clarity TM Western ECL substrate Bio-Rad cat#170–5060) according to the manufacturer’s recommendation. ImageJ analysis software was used to analyze the band concentration of the aim proteins against β-actin (internal reference) by protein normalization on the ChemiDoc MP imager.

### Histopathological studies of the kidneys

Renal tissue was fixed in 10% neutral buffered formaldehyde and then dehydrated in rising grades of alcohol, clear in xylene, and fixed in paraffin wax. Paraffin Sects. (4 µm) were stained with hematoxylin and eosin (H&E) and PAS special stain for histopathological analysis.

### Immuno-histochemical study and quantitative morphometric analysis of caspase-3 and LC3 II in kidney tissues

After mounting tissue pieces (5 μm thick) on positively charged slides, they were allowed to cure at 37 °C for 30 min. After deparaffinization, sections were recovered using high and low PH EnVision FLEX antigen retrieval solutions in a Dako PT link device (at 97 °C for 20 min). Dako Autostainer Link 48 was used for immunohistochemistry. A peroxidase blocking solution was used, then the sections were incubated for 30 min with the primary antibodies, rabbit polyclonal anti‐caspase‐3 (1:1000 dilution; BD Biosciences) and rabbit monoclonal anti-LC3II (0.5 mg/ml, 1:400; Bio-Rad, Alfred Nobel Drive, Hercules, CA, USA). After a 20-min incubation with horseradish peroxidase polymer, the chromogen diaminobenzidine (DAB) was added to detect the immune response. The sections were counterstained with Mayer’s hematoxylin and morphometrically analyzed. Slides were photographed by an Olympus® digital camera installed on Olympus® microscope with 1/2 X photo adaptor, using 10X objective. The result images were analyzed on Intel® Core I7® based computer using Video Test Morphology® software (Russia) with a specific built-in routine for area, % area, measurement, object counting, and contact_Angle.

The image analysis system was done by image analysis software program (Image J. version 1.48). The nuclear caspase-3 immuno-reactivity was assessed semi-quantitatively by determining the percentage of cells exhibiting positive staining: 10% staining was classified as positive (Ok Atılgan et al. 2023). Assessment of LC3 II expression with IHC was classified as negative (if staining was 0%), class 1 (if staining was 0–5%), class 2 (if staining was 5–35%), class 3 (if staining was 35–65%), and class 4 (if staining was 65–100%). Expression of LC3 II positive is LC3 II with a class 4 score (Andreas et al. 2023).

### Electron microscopy of changes in mitochondrial morphology

Renal samples were fixed in 2% phosphate-buffered glutaraldehyde, washed three times in PBS and fixed in 1% phosphate-buffered osmium tetroxide, and thereafter dehydrated in rising grades of ethanol and embedded in epoxy resin. Ultrathin Sects. (40–50 nm in thickness) were obtained with a Leica ultratome (Ultracut UCT; Leica Microsystems GmbH, Vienna, Austria) and stained with uranyl acetate and lead citrate (Bozzola and Russel 1999; Kuo 2007). Sections were examined and photographed with JEOL-100 JEM (Jeol, Tokyo, Japan) in the Electron Microscopic Unit of the Faculty of Medicine, Tanta University.

### Statistical analysis

Data processing and statistical analysis were performed using the SPSS software version 21 (SPSS Inc., Chicago, IL). Baseline characteristics are presented as mean ± SD. The significance level was determined by one-way analysis of variance (ANOVA) for multiple groups, followed by post hoc analysis Tukey’s multiple comparison test. A difference is considered to be statistically significant when *P* < 0.05.

## Results

### Effect of artemisinin on serum renal functions

Three days of DIC administration exhibits renal injury in rats, as demonstrated by a notable increment of serum creatinine and urea levels as compared to the control groups. Conversely, ART treatment in both prophylactic and therapeutic groups resulted in a remarkable decline in their levels relative to DIC group (Table 1).

**Table 1:** Effect of artemisinin on serum renal functions

Parameters	Group I	Group II	Group III	Group IV	Group V
Serum creatinine (mg/dl)	0.65 ± 0.01	0.60 ± 0.02	1.11 ± 0.12^*$^	0.77 ± 0.11^**$#**^	0.86 ± 0.08^*$#^
Serum urea (mg/dl)	25.33 ± 1.86	16 ± 2.68	78.67 ± 10.48^*$^	43.50 ± 6.95^*$#^	53 ± 7.16^*$#^

Data are expressed as mean ± SD

*Group I* negative control group, *Group II* artemisinin control group, *Group III* diclofenac group, *Group IV* diclofenac + artemisinin prophylactic group, *Group V* diclofenac followed + artemisinin therapeutic group

*p < 0.05 compared to Group I

^$^*p* < 0.05 compared to Group II

^#^*p* < 0.05 compared to Group III

### Effect of artemisinin on oxidative stress-related markers

**Fig. 1:**
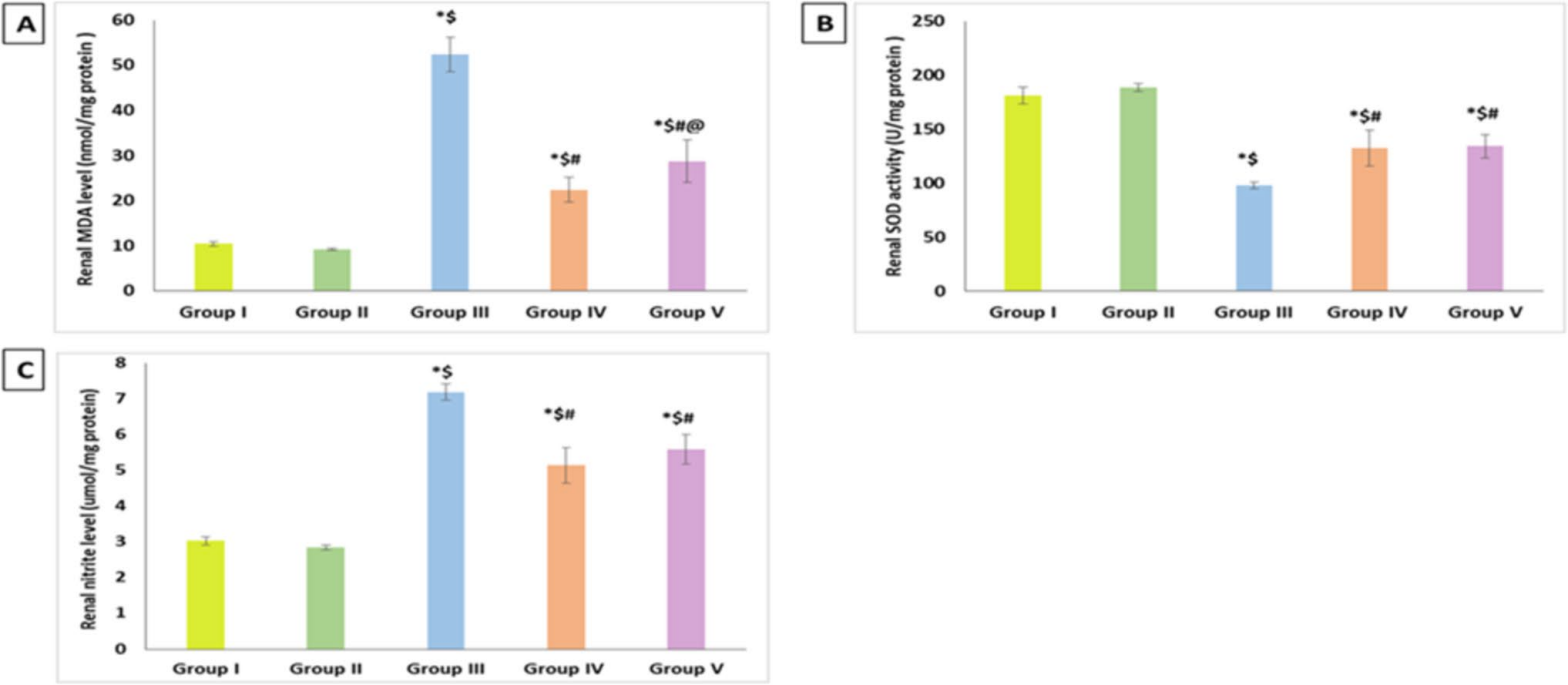
Effect of Artemisinin on renal levels of **A** MDA, **B** SOD activity, and **C** nitrite. Data are expressed as mean ± SD. *p < 0.05 compared to Group I, ^$^*p* < 0.05 compared to Group II, ^#^*p* < 0.05 compared to Group III, and ^@^*p* < 0.05 compared to Group IV. Group I, negative control group; Group II, artemisinin control group; Group III, diclofenac group; Group IV, diclofenac + artemisinin prophylactic group; and Group V, diclofenac followed + artemisinin therapeutic group

DIC treatment significantly elevated renal MDA levels in comparison to the control groups while concurrently significantly diminishing renal SOD activity. When compared to the DIC group, the administration of ART significantly reversed these alterations in both the prophylactic and therapeutic groups. Additionally, compared to the ART therapeutic group, the ART prophylactic group showed a significant decrease in renal MDA levels (Fig. 1A, B).

### Effect of artemisinin on renal nitrites

As illustrated in Fig. 1C, in comparison to the control groups, DIC administration showed a notable rise in nitrite levels in renal homogenates. When compared to the DIC group, the ART group exhibited a notable decline in its level in both the prophylactic and therapeutic groups.

### Effect of artemisinin on mitochondrial related markers (PGC-1α, Drp1, and mitochondrial ATP)

**Fig. 2:**
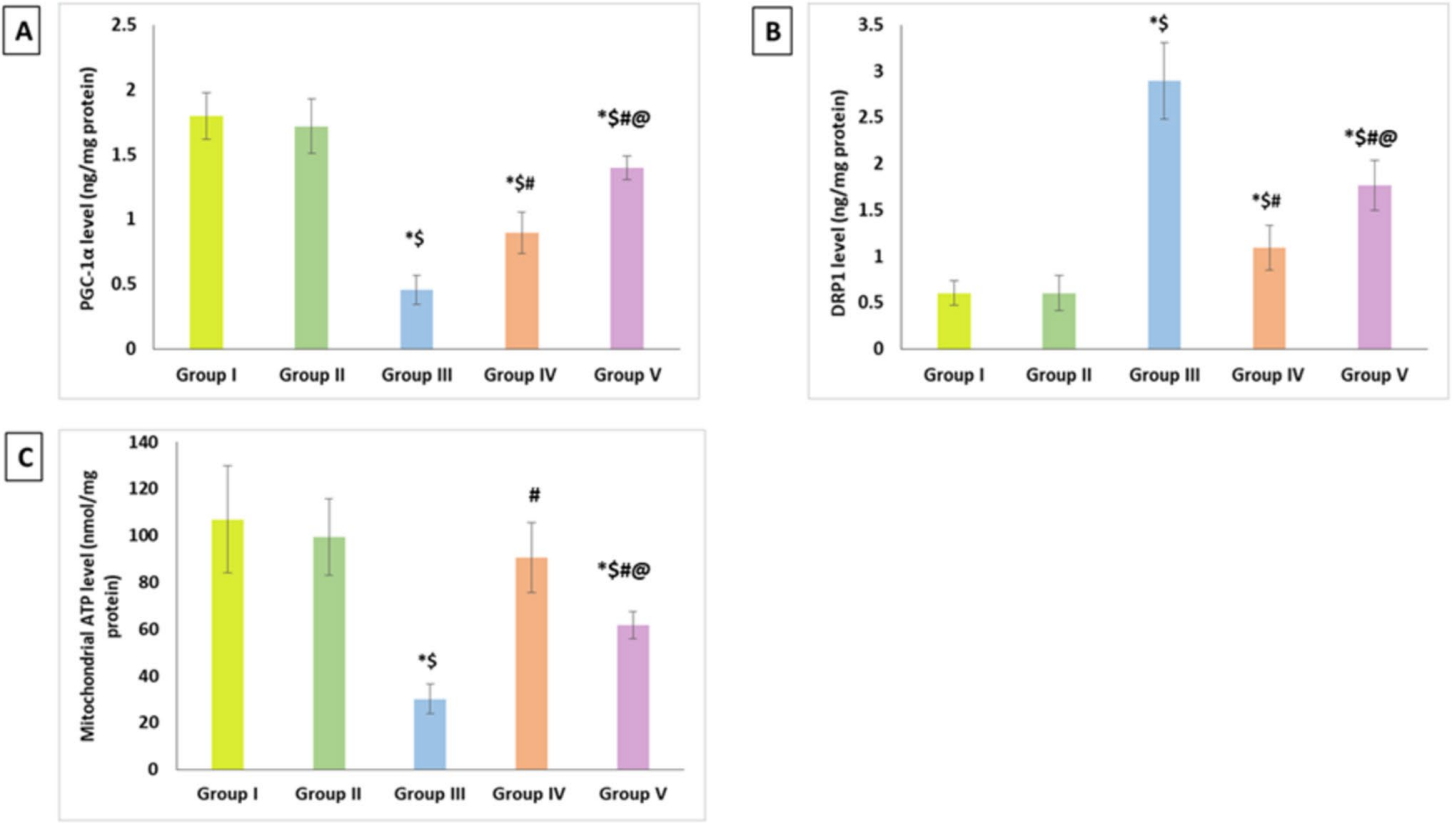
Effect of artemisinin on mitochondrial related markers **A** PGC-1α, **B** Drp1, and **C** mitochondrial ATP. Data are expressed as mean ± SD. *p < 0.05 compared to Group I, $*p* < 0.05 compared to Group II, ^#^*p* < 0.05 compared to Group III, and ^@^*p* < 0.05 compared to Group IV. Group I, negative control group; Group II, artemisinin control group; Group III, diclofenac group; Group IV, diclofenac + artemisinin prophylactic group; and Group V, diclofenac followed + artemisinin therapeutic group

Since renal mitochondria play a crucial role in regulating renal functions, the current study assessed several mitochondrial functions to evaluate the renal mitochondrial quality. Immunoassay PGC-1α levels was used measure mitochondrial biogenesis, and the results displayed a notable decline in the DIC group compared to the control groups. However, compared to the DIC group, both ART-treated groups showed a significant increment in their level. When compared to the ART therapeutic group, the PGC-1α level in the ART prophylactic group increased significantly (Fig. 2A).

In order to assess mitochondrial dynamics, Drp1 was measured. We discovered that whereas ART significantly diminished Drp1 levels in both groups, with a significant decline in the prophylactic group relative to the therapeutic group, DIC treatment significantly increased Drp1 as compared to the control groups (Fig. 2B).

Using ATP level, the bio-energetic potential of the renal mitochondria was evaluated. Compared to the control groups, the DIC group showed a notable drop in mitochondrial ATP; however, ART significantly reversed this impairment in both groups. Additionally, the prophylactic ART group had a notable elevation in ATP level in comparison to the therapeutic group (Fig. 2C).

### Effect of artemisinin on SIRT3 relative mRNA gene abundance and protein expression

**Fig. 3:**
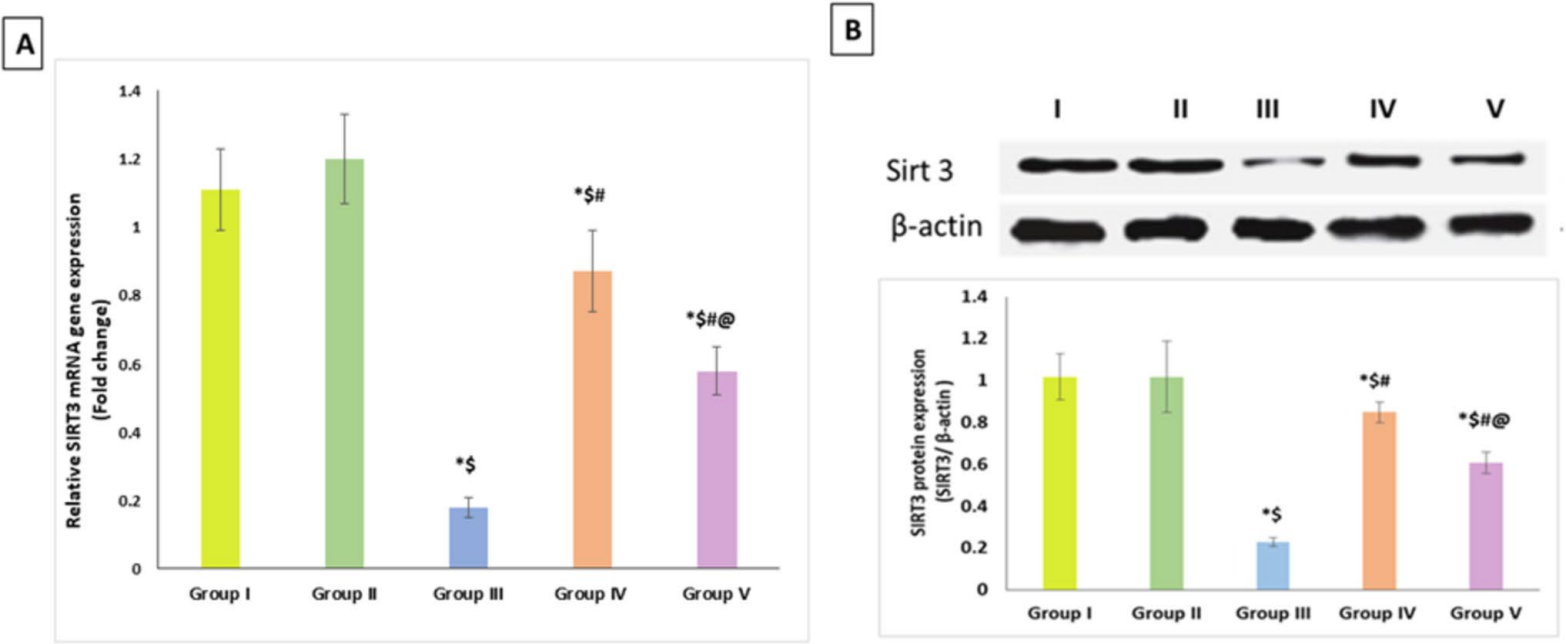
Effect of artemisinin on **A** relative SIRT3 mRNA gene expression and **B** SIRT3 protein expression. Data are expressed as mean ± SD. *p < 0.05 compared to Group I, ^$^*p* < 0.05 compared to Group II, ^#^*p* < 0.05 compared to Group III, and ^@^*p* < 0.05 compared to Group IV. Group I, negative control group; Group II, artemisinin control group; Group III, diclofenac group; Group IV, diclofenac + artemisinin prophylactic group; and Group V, diclofenac followed + artemisinin therapeutic group

Compared to the control groups, DIC administration revealed a significant decrement of SIRT3 mRNA gene expression about 0.18 fold change relative its expression in the control group (by 82%, *p* < 0.001). These changes where counteracted by ART where gene expression of SIRT3 was significantly ameliorated in both groups compared to the DIC group; the prophylactic ART improved SIRT3 expression about 0.87 fold changes relative to the control group (by 13%, *p* < 0.05); however, therapeutic ART mitigated SIRT3 expression about 0.58 fold changes to its expression in the control group (by 42%, *p* < 0.001). Additionally, ART prophylactic group exhibited significant upregulation of SIRT3 mRNA as compared to the therapeutic group (Fig. 3A). However, Western Blotting of the SIRT3 protein expression was carried out for additional validation, and the results showed that SIRT3 expression was significantly downregulated in the DIC group compared to the control groups. As opposed to the DIC and control groups, ART dramatically reduced SIRT3 protein expression. In addition, it was noted that ART prophylactic group exhibited significant difference when compared to the therapeutic group, as shown in Fig. 3B.

### Histopathological studies of the kidneys

**Fig. 4:**
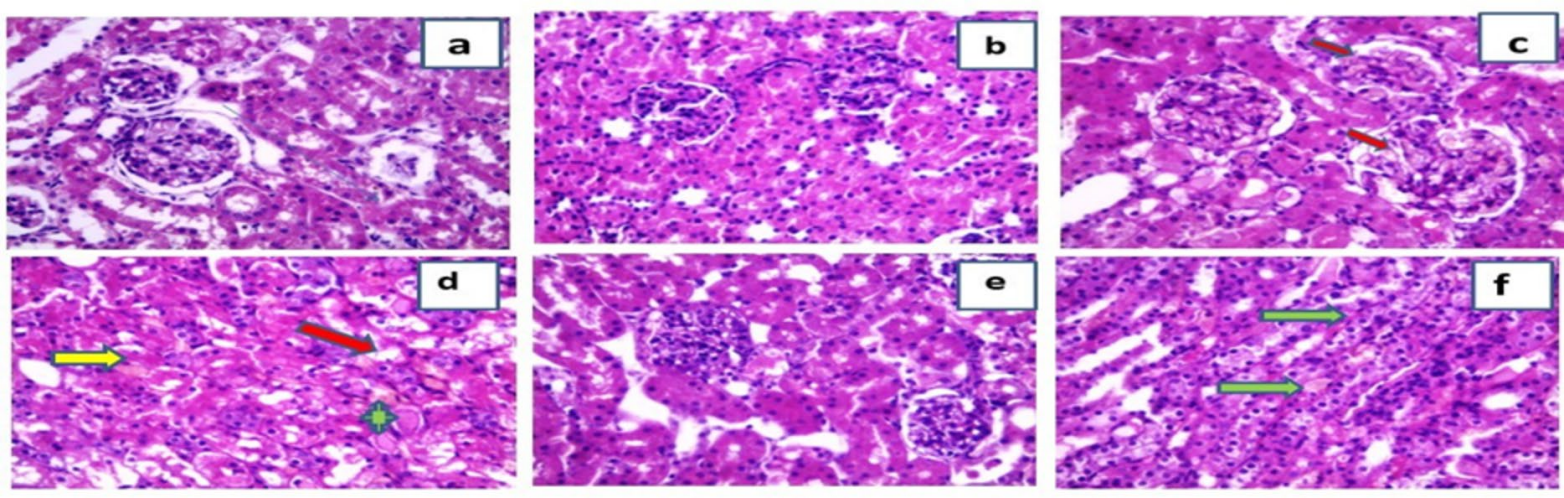
Effect of artemisinin on histopathological alterations (H&E × 400) in different groups. **a** Control group showing normal renal histology. **b** Artemisinin control group showing near normal renal histology. **c** Diclofenac group showing glomerular congestion (red arrows). **d** Diclofenac group showing tubular cell apoptosis (yellow arrow), vacuolation (red arrow), and intraluminal casts (green asterisk). **e** Diclofenac + artemisinin prophylactic group showing healthy tubular cells and less intraluminal casts. **f** Diclofenac followed + artemisinin therapeutic group showing less glomerular congestion

Examination of H&E stained slides form both the control and ART control groups exposed normal kidney tissue with healthy glomeruli and tubules (Fig. 4a, b). However, examination of kidney tissues from the DIC group revealed major changes in histological structure of the kidney. Renal tissues showed congestion of the glomerular capillaries and many apoptotic cells in the renal tubules. Also, some hyaline casts appear in the lumina of renal tubules and some inflammatory cells besides vacuolation in the lining tubular epithelial cells (Fig. 4c, d). On the other side, renal tissues from the ART prophylactic group showed major improvement of the renal histology meaning that less apoptotic cells were present and the hyaline casts disappeared. Moreover, glomerular capillary congestion was too much decreased and less vacuolation of the epithelial cells lining the tubules (Fig. 4e) while renal tissues from the ART therapeutic group revealed moderate improvement of the renal tissue histology which means decreased number of apoptotic bodies were present and the hyaline casts focally disappeared. Besides, glomerular capillary congestion was also decreased with less vacuolation of the epithelial cells lining the tubules (Fig. 4f).

**Fig. 5:**
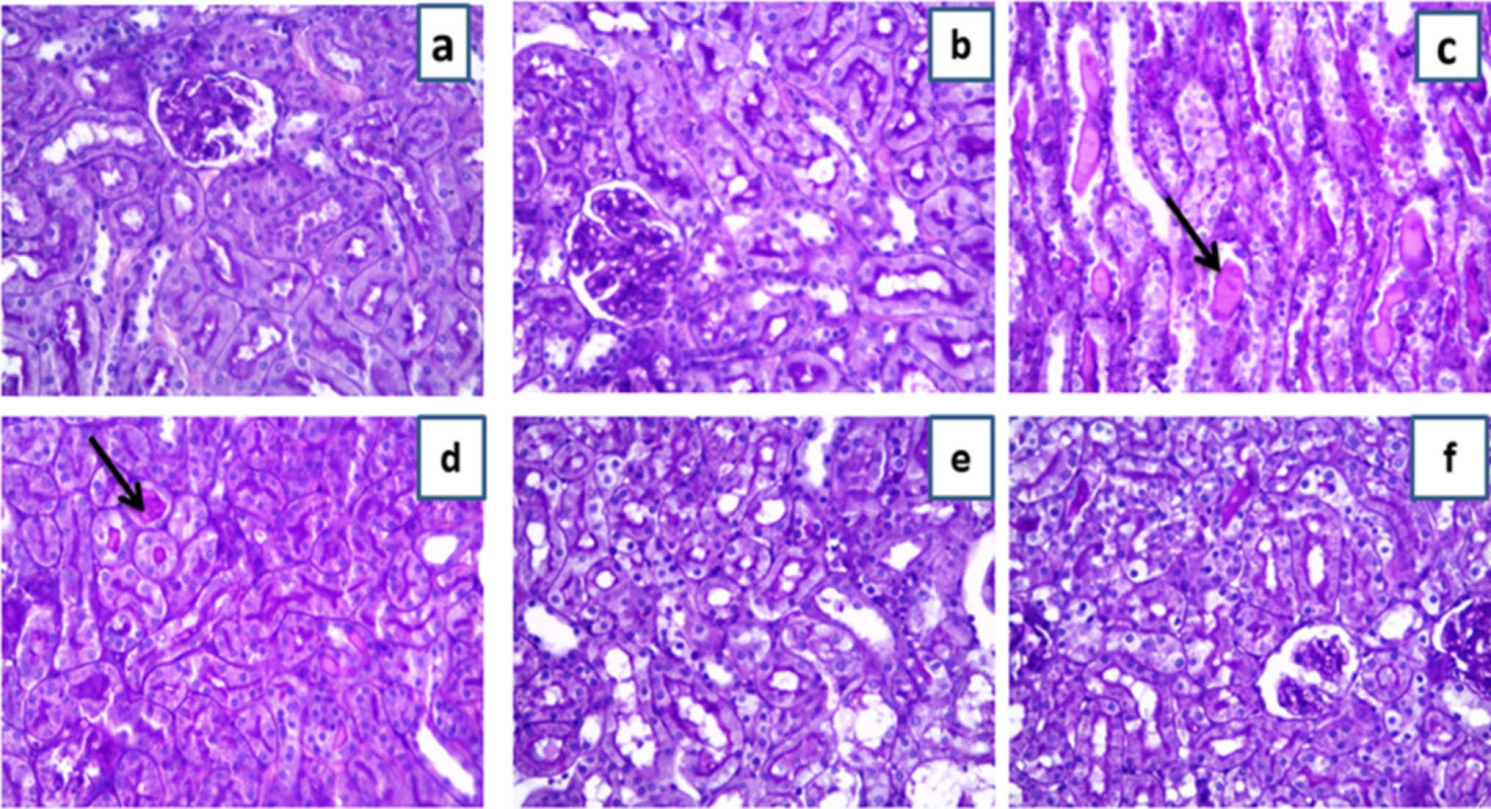
Representative photomicrograph of renal tissues stained with PAS special stain (× 400). **a** Control group and **b** artemisinin control group showing normal thickening of the basement membranes and no PAS-positive intracellular accumulation or intratubular luminal casts. **c, d** Diclofenac group showing PAS positive intraluminal tubular casts (black arrows) and intracellular PAS-positive granules. **e** Diclofenac + artemisinin prophylactic group showing less intraluminal PAS-positive casts. **f** Diclofenac followed + artemisinin therapeutic group showing less intraluminal PAS-positive casts

Additionally, histopathological examination of PAS stained slides from the control and ART control groups revealed normal kidney tissue without any PAS positive accumulation or intratubular casts and normal thickness of the basement membranes (Fig. 5a, b). Examination of sections from the DIC-treated group revealed intratubular PAS-positive casts. Also, thickening of the glomerular and tubular basement membranes was noticed (Fig. 5c, d). The ART prophylactic group revealed major improvement of the kidney structure in the form of disappearance of the tubular intraluminal casts (Fig. 5e). However, the ART therapeutic group revealed moderate improvement of the kidney structure as the tubular intraluminal casts focally disappeared (Fig. 5f). These results indicated that ART is more effective when used as a prophylactic drug than a therapeutic one.

### Immuno-histochemical results

#### Effects of artemisinin on caspase-3 immunoreactivity in renal tissues

**Fig. 6:**
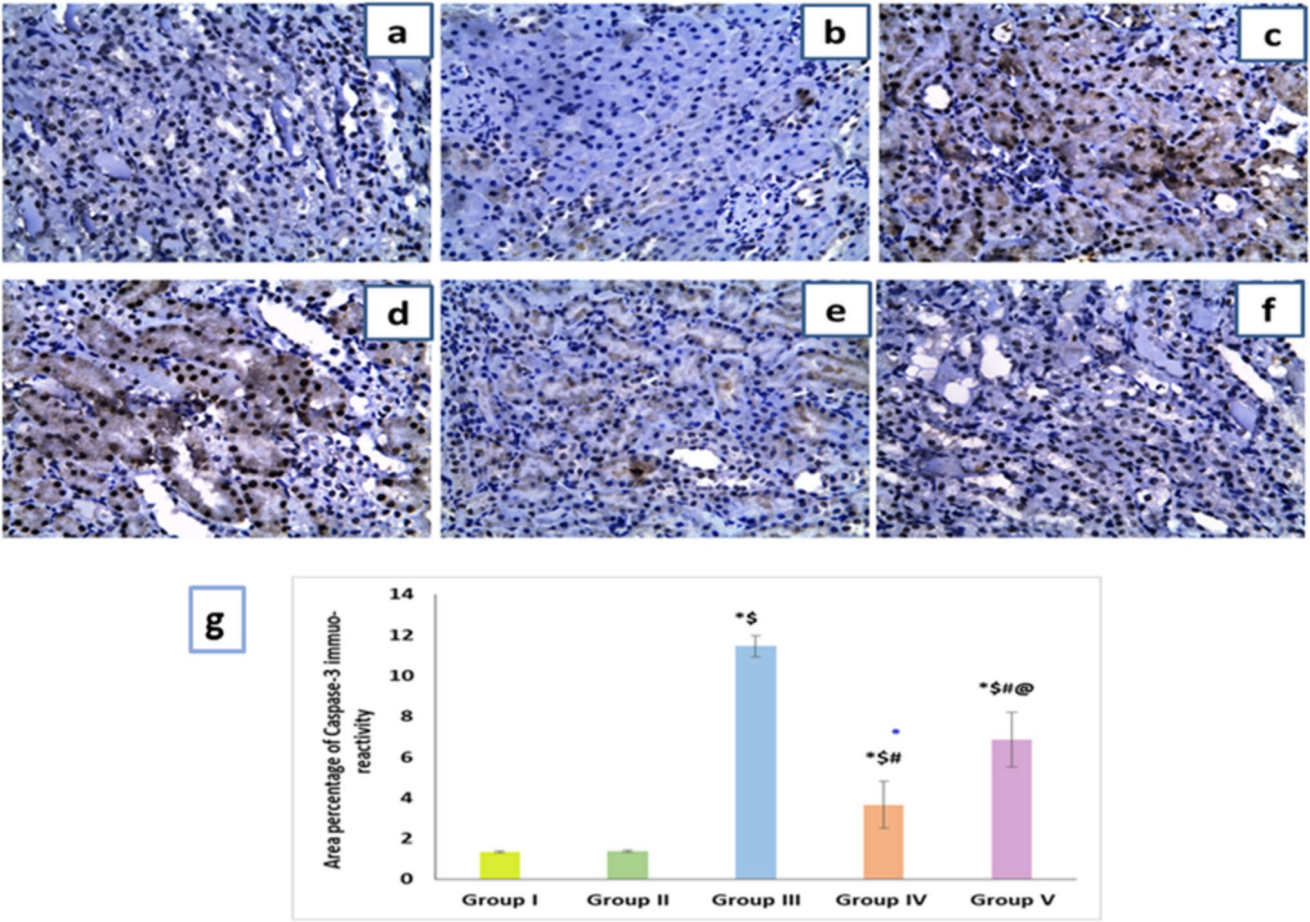
Effect of artemisinin on caspase-3 immunoreactivity in different groups (× 400). **a** Control group and **b** artemisinin control group showing negative nuclear expression of caspase-3. **c, d** Diclofenac group showing positive nuclear expression in more than 10% of tubular epithelial cells. **e** Diclofenac + artemisinin prophylactic group showing negative nuclear expression. **f** Diclofenac followed + artemisinin therapeutic group showing negative nuclear expression. **g** A morphometric analysis of the mean area percentage of caspase-3 immune-reactivity. Data are expressed as mean ± SD. *p < 0.05 compared to Group I, ^$^*p* < 0.05 compared to Group II, ^#^*p* < 0.05 compared to Group III, and ^@^*p* < 0.05 compared to Group IV. Group I, negative control group; Group II, artemisinin control group; Group III, diclofenac group; Group IV, diclofenac + artemisinin prophylactic group; and Group V, diclofenac followed + artemisinin therapeutic group

As shown in Fig. 6a, b, negative nuclear expression of caspase-3 was noticed in both the control and ART control groups. However, the DIC-treated group showed a strong caspase-3 expression in renal tissues when compared to both control groups (Fig. 6c, d). Caspase 3 nuclear expression started to decline in both ART-treated groups; however, the ART prophylactic group showed more decrease of caspase 3 nuclear expression denoting that ART has a very effective role in protection of the kidney against the toxic effect of DIC through lowering apoptosis of renal tubular cells, and this protective effect is more obvious when administered as a prophylactic drug than a therapeutic one (Fig. 6e, f). The statistical analysis of morphometric results displayed notable elevation in caspase-3 positive immunoreaction percentage area in the DIC-treated group compared to the both control groups. Conversely, the area of percentage was altered in both ART-treated groups when compared to DIC group. Notably, the ART prophylactic group showed significant decline in caspase-3 positive immunoreaction percentage area when compared to the therapeutic group.

#### Effects of artemisinin on LC3 II immunoreactivity in renal tissues

**Fig. 7:**
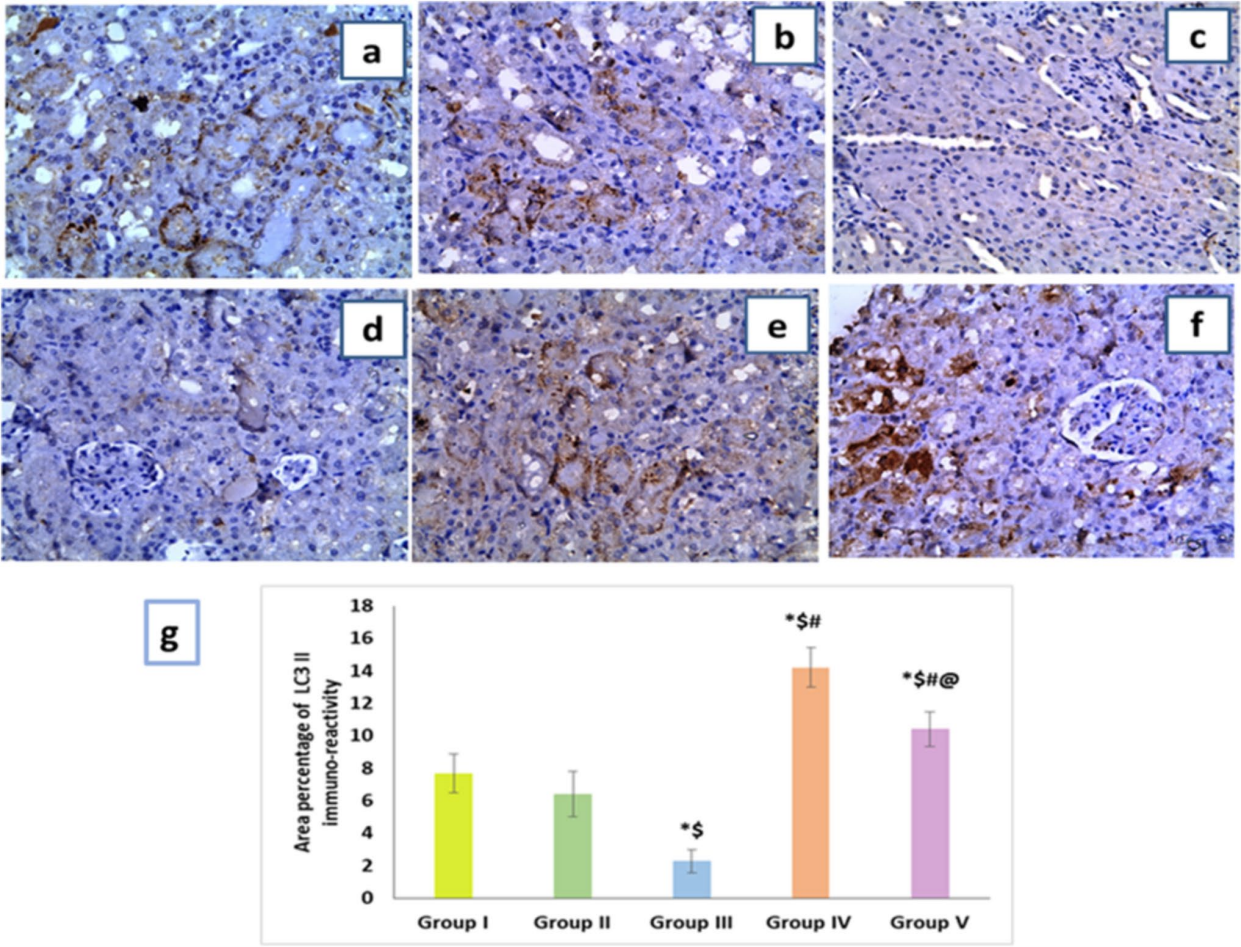
Effect of artemisinin on LC3 II immunoreactivity in different groups (× 400). **a** Control group and **b** artemisinin control group showing positive cytoplasmic expression class 2. **c, d** Diclofenac group showing negative cytoplasmic expression class 0. **e** Diclofenac + artemisinin prophylactic group showing positive cytoplasmic expression class 4. **f** Diclofenac followed + artemisinin therapeutic group showing positive cytoplasmic expression class3. **g** A morphometric analysis of the mean area percentage of LC3 II immune-reactivity. Data are expressed as mean ± SD. *p < 0.05 compared to Group I, ^$^*p* < 0.05 compared to Group II, ^#^*p* < 0.05 compared to Group III, and ^@^*p* < 0.05 compared to Group IV. Group I, negative control group; Group II, artemisinin control group; Group III, diclofenac group; Group IV, diclofenac + artemisinin prophylactic group; and Group V, diclofenac followed + artemisinin therapeutic group

As shown in Fig. 7a, b, cytoplasmic expression of LC3 II in both control groups demonstrated that LC3 II was class 2 showing many cells with strong positive immunoreaction of LC3 II. However, the DIC-treated group revealed the lowest score class 0 with negative reaction a LC3II immunoreaction (Fig. 7c, d). On the other side, sections from both ART prophylactic and therapeutic groups showed score class 4 and 3, respectively, with frequent cells of strong positive immunoreaction (Fig. 7e, f). Furthermore, the statistical analysis of morphometric of the mean percentage area of positive LC3 II immunoreaction results displayed notable decline in the area percentage of immunoreaction in the DIC group as compared to both control groups. However, compared to the DIC-treated group, there was a notable elevation in LC3 II immunoreaction in both ART groups with a significant increase in the prophylactic group denoting that ART has a very effective role in protection of the kidney against the toxic effect of DIC by ameliorating mitochondrial autophagy.

#### Effect of artemisinin on mitochondrial morphological changes detected by electron microscopy

**Fig. 8:**
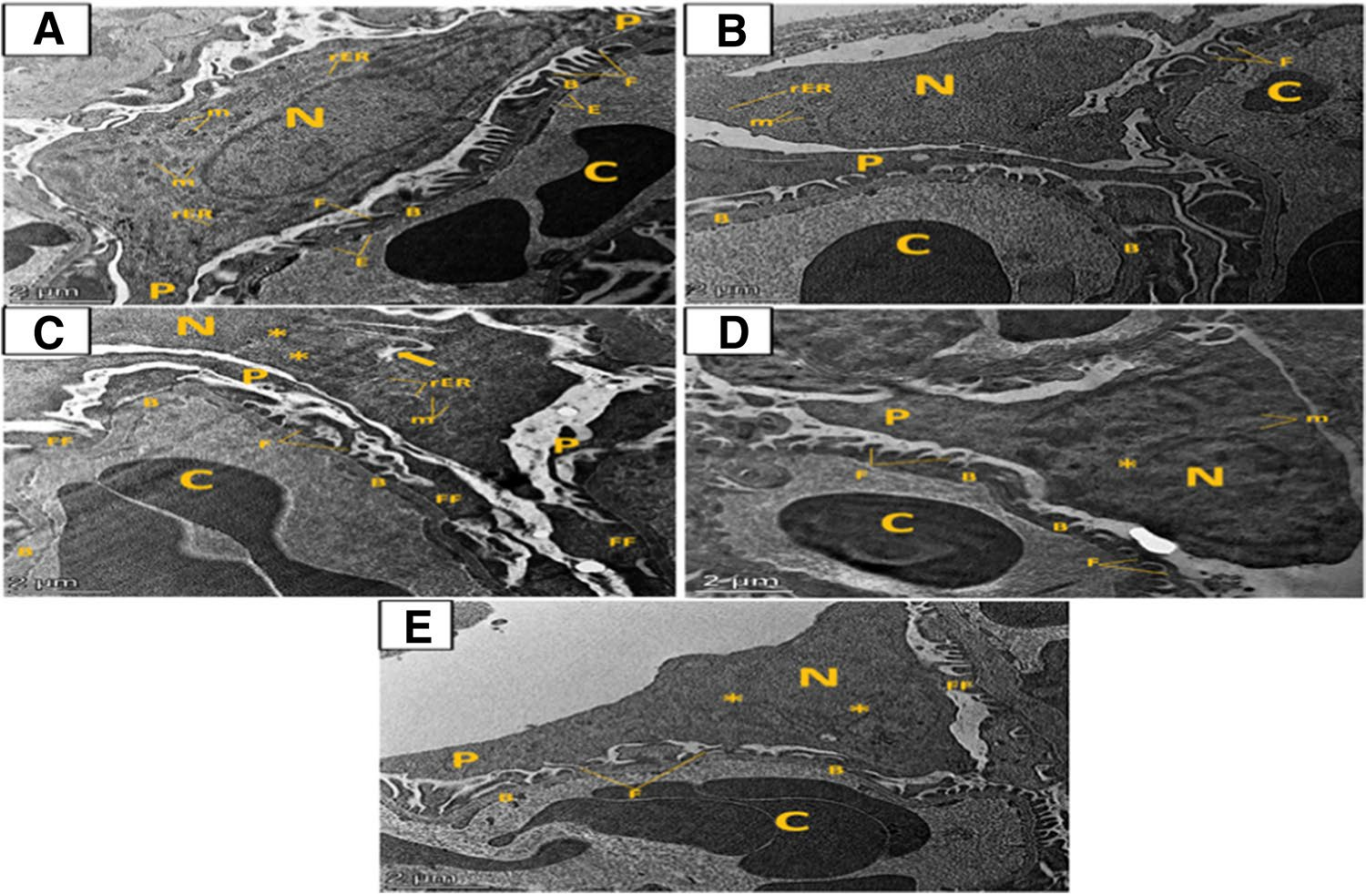
Transmission electron micrographs of ultrathin sections of the renal cortex of different groups. **A** Control group and **B** artemisinin control group, showing normal renal corpuscles formed of glomerular blood capillaries (C) lined by fenestrated endothelium (E) surrounded with podocytes. The podocytes had rounded euchromatic nuclei (N) and their cytoplasm was rich in mitochondria (m) and rough endoplasmic reticulum (rER). From the podocytes’ cell bodies arised primary processes (P) and multiple secondary foot processes (F) which rested on a regular basement membrane (B). **C** Diclofenac group showed a glomerular capillary (C) with glomerular basement membrane (B) thickening. The podocytes had nuclei (N) with irregular outline (*) and their cytoplasm shows perinuclear spacing (arrow) and apparent dilated cisternae of rough endoplasmic reticulum (rER). Fused foot processes (FF) and apparent thinning of the primary process (P) are seen. However, **D** diclofenac + artemisinin prophylactic group and **E** diclofenac followed + artemisinin therapeutic group showed glomerular capillaries with nearly normal glomerular basement membrane (B) in prophylactic group (Fig. 8D) and minimal glomerular basement membrane thickening in therapeutic group (Fig. 8E). The podocytes cytoplasm are rich in mitochondria (m) and their primary processes (P) are nearly normal however they have indented nuclei (N) and group IV showed minimal fused secondary processes (FF) (Fig. 8E)

Ultra-thin sections of the renal cortex of animal groups showed normal renal corpuscles in both the control and ART control groups formed of glomerular blood capillaries lined by fenestrated endothelium surrounded with podocytes. The podocytes had rounded euchromatic nuclei and their cytoplasm was rich in mitochondria and rough endoplasmic reticulum. The podocytes’ cell bodies arised primary processes and several secondary processes (foot processes) that rested on an intact basement membrane (Fig. 8A, B). However, examination of DIC group shown a glomerular capillary with glomerular basement membrane thickening. The podocytes had irregular nuclei, and their cytoplasm showed perinuclear spacing and apparent dilated cisternae of rough endoplasmic reticulum. Fused foot processes and apparent thinning of the primary process were also appeared (Fig. 8C). Electron microscopic examination of the ART prophylactic group exposed glomerular capillaries with nearly normal glomerular basement membrane, however, there is minimal glomerular basement membrane thickening in the ART therapeutic group. Furthermore, the podocytes cytoplasm were rich in mitochondria and their primary processes were nearly normal and had indented nuclei in the ART prophylactic group; however, the ART therapeutic group showed minimal fused secondary processes (Fig. 8D, E).

**Fig. 9:**
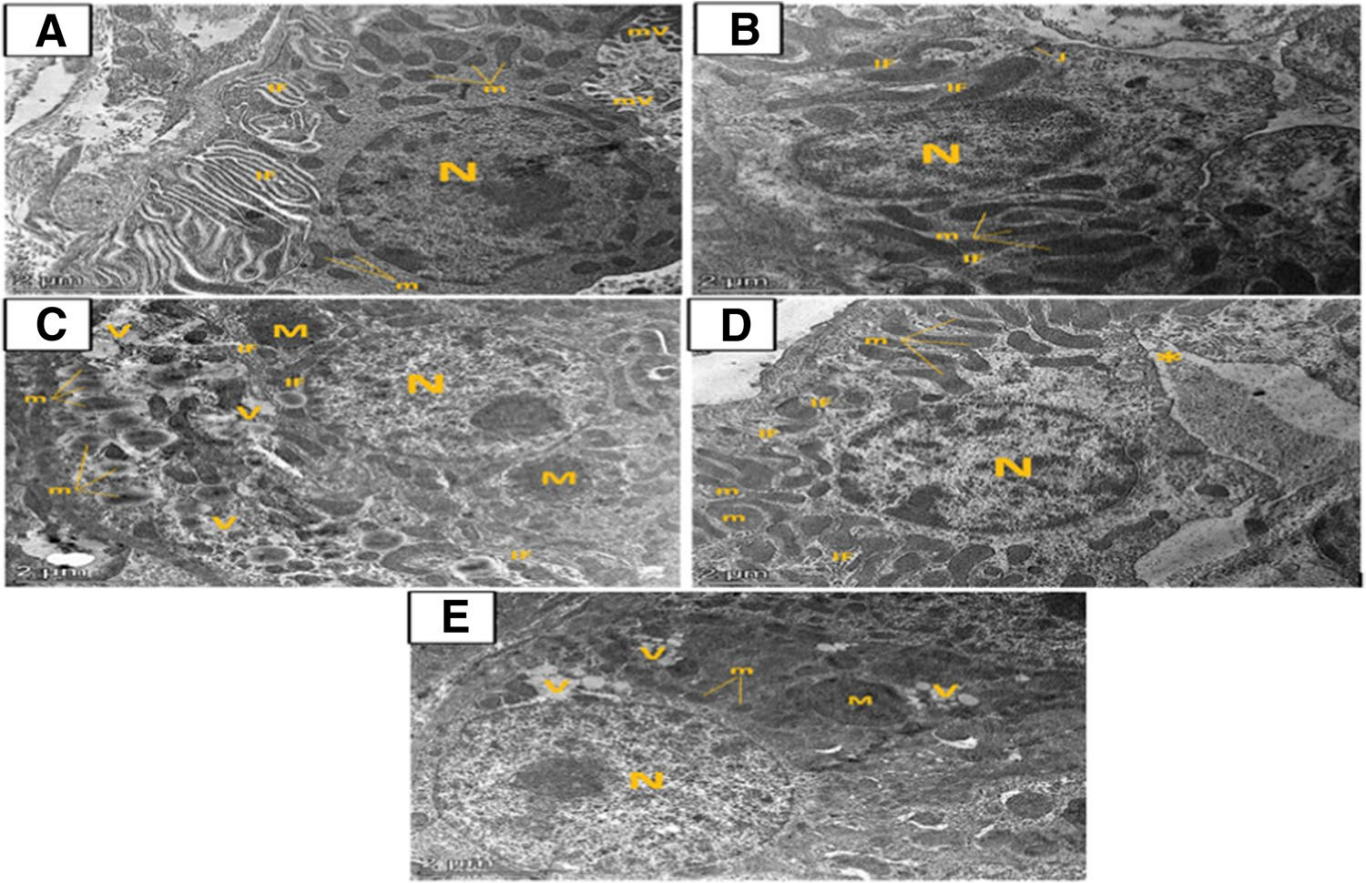
Transmission electron micrographs of ultrathin sections of the renal cortex of different groups. **A** Control group and **B** artemisinin control group showing normal distal convoluted tubular cells with rounded euchromatic nuclei (N) and their cytoplasm are rich in rounded and elongated basely located mitochondria (m). There are many apical microvilli (mV) and a tight junction (J) of the plasmalemma of adjoining cells is seen. Numerous organized basal infoldings (IF) of the cell membrane are noticed. Ultrathin sections of the distal convoluted tubular cells in **C** diclofenac group showed disorganization of basal infoldings (IF), their cytoplasm is vacuolated (V) and contains numerous vacuolated mitochondria (m) with destroyed cristae as well as hypertrophied giant mitochondria (M). However, ultrathin sections of the distal convoluted tubular cells in **D** diclofenac + artemisinin prophylactic group and **E** diclofenac followed + artemisinin therapeutic group appeared less affected with normal rounded euchromatic nuclei (N) and numerous normal elongated basely located mitochondria (m) in between organized basal infoldings (IF) of the cell membrane. However, in ultrathin sections of prophylactic group, the distal tubular cells are noticed to be widely separated (*) with less apical microvilli, on the other hand, sections of the therapeutic group show some cytoplasmic vacuoles (V) as well as few hypertrophied giant mitochondria (M)

On the other hand, both control groups showed normal distal convoluted tubular cells with rounded euchromatic nuclei, and their cytoplasm were rich in rounded and elongated basely located mitochondria. There were many apical microvilli and a tight junction of the plasmalemma of adjacent cells was seen. Numerous organized basal infoldings of the cell membrane were noticed (Fig. 9A, B). An electron microscopic examination of the distal convoluted tubular cells in DIC-treated group showed inadequacy of basal infoldings; their cytoplasm was vacuolated and contained numerous vacuolated mitochondria with destroyed cristae as well as hypertrophied giant mitochondria (Fig. 9C). The distal convoluted tubular cells in both ART groups (prophylactic and therapeutic) appeared less affected with normal rounded euchromatic nuclei and numerous normal elongated basely located mitochondria in between organized basal infoldings of the cell membrane. However, the therapeutic group showed some cytoplasmic vacuoles as well as few hypertrophied giant mitochondria. The distal tubular cells were noticed to be widely separated with less apical microvilli (Fig. 9D, E).

## Discussion

Kidney injury is important target for annoying clinical effects related to the use of NSAIDs (Sivaraj and Umarani 2018). DIC-induced nephrotoxicity has been linked to mechanisms such as mitochondrial malfunction and increased oxidative stress, which are primarily caused by DIC's metabolism to its active metabolites (Nouri and Heidarian 2019b). The current research revealed the novel evidence regarding the protective effects of ART on DIC-induced nephrotoxicity through restoring mitochondrial homeostasis and regulating oxidative stress via SIRT3 activation as a promising therapeutic strategy to cope with DIC-induced renal injury.

The current data revealed that DIC administration in rats caused abnormal kidney functions represented by increased both serum Cr and urea levels, marked decrease in SIRT3 expression which triggered oxidative stress associated with declined antioxidant levels, disturbed mitochondrial homeostasis as showed by decreased PGC-1α and mitochondrial ATP concomitant with increased Drp1 levels, with significant dysregulation of autophagy and apoptosis. However, ART administration reversed these alterations. All these changes were confirmed by histopathological and immune-histochemical analyses.

Evaluation of renal function was displayed in this study by measuring serum Cr and urea. This study confirmed that DIC caused a notable elevation in serum Cr and urea levels compared to the control groups. This effect agreed with the preceding results of Abiola et al. (2019). ART administration in both prophylactic and therapeutic groups in our study showed significant reduction in serum Cr and urea compared to the DIC group, and this result was in agreement with Feng et al.’s (2022) and Han et al.’s (2021) studies. It has been reported that ART improved renal function by several mechanisms such as by inhibiting vascular endothelial growth factor (Miao et al. 2022) or inhibiting TNF-α and maintaining occludin expression or through elevation of vascular endothelial cadherin expression in endothelial cells (Cheng et al. 2018, Li et al. 2018).

In our study, DIC administration significantly decreased SIRT3 gene expression and its protein expression in renal tissues of rats which was in concomitant with the result of Zhao et al (2018) and Locatelli et al. (2020). ART administration either in prophylactic or therapeutic groups significantly reversed SIRT3 expression, this result is similar to previous study Han et al (2021). According to previous studies, dihydroartemisinin may have the ability to increase SIRT3 activity in animal models (Zhang et al. 2022). To the best of our knowledge, this represents the first study that demonstrated the beneficial effects of ART treatment on DIC-induced renal injury via regulation of SIRT3.

Oxidative stress is a vital mediator in the development and progress of renal injury (Ruiz et al. 2013). Mitochondrial ROS is increased under induced kidney injury, hypoxia, toxicity drug and pathologic situation. The mitochondria damage associated with overproduction of ROS, has been identified to play a major role in oxidative stress and cell death (Amini et al. 2022). Excessive ROS can predispose proteins, lipids and DNA to oxidative damage (Amini et al. 2024) Disruption of the kidney redox state stimulates fibrogenic pathways and ultimately renal failure (Okamura and Pennathur 2015). Given these reasons, it is reasonable to assume that perhaps the ameliorative effect of ART could be due to its anti-oxidant properties and this agent could protect the renal tissue from DIC-induced free radicals.

In our study, DIC disturbed the kidney redox state and reduced the antioxidant enzymes activity in the renal tissue (Nouri and Heidarian 2019b). DIC administration significantly elevated both renal MDA and NO levels with concomitant significant decline of renal SOD activity compared to the control groups which was in line with other studies (Ogbe et al. 2019; Nouri and Heidarian 2019a; Nouri et al. 2021; Abiola et al. 2019). On the other hand, ART administration either prophylactic or therapeutic significantly reversed these alterations, in agreement with the results of An et al.’s (2017) and Liu et al.’s (2019) studies. ART antioxidant activity could be attributed to SIRT3 via increasing the ETC’s efficiency through the deacetylation of complexes I, III, and IV, which decreases electron leakage and ROS generation.In addition, SIRT3 deacetylates SOD2 to increase its activity and deacetylates p53 to inhibit the interaction between SOD2 and p53, which increases SOD2’s antioxidant activity (Fan et al. 2021; Ouyang et al. 2019).

As the mitochondria are the dynamo of the cells and contribute in several cellular processes, its impairment plays an essential role in kidney damage (Galvan et al. 2017). Preserving structural integrity of mitochondrial and improving mitochondrial biogenesis can help kidney salvage from renal injury (Whitaker et al. 2016; Szeto et al. 2017). PGC-1α is a principal controller of mitochondrial biogenesis and antioxidant effects and as well, it is included in the mitochondrial quality regulation that includes dynamics of mitochondrial network and autophagy elimination of spoiled mitochondria (Halling and Pilegaard 2020). DIC administration causes significant decrease PGC-1α level; this was in agreement with Tran et al. (2011) study. The level of PGC-1α was significantly elevated after ART administration either in prophylactic or therapeutic groups, this is in agreement with the result of Wang et al. (2019) study.

Mitochondrial dynamics distraction, mainly regulated by two opposite processes: fission and fusion, plays a critical role in kidney injury; mitochondrial fragmentation cause cytochrome C release and apoptotic cell death (Lyamzaev et al. 2022). In our study, DIC administration significantly increased Drp1 levels as compared to the control groups; this was the same with the result of Perry et al. (2018). Increased expression of Drp 1 induces mitochondrial fission (Funk and Schnellmann 2012). The balance between fission and fusion edicts mitochondrial shape. Fusion is linked with increased ATP synthesis, while fission is linked with diminished oxidative phosphorylation and ROS production (Wai and Langer 2016). Herein, ART administration in either prophylactic or therapeutic groups produced significant decrease Drp1 levels as compared to DIC group This is in concomitant with Qin et al. (2022) who illustrated that ART could reduce mitochondrial ROS production, delay mitochondrial fission, restoring mitochondrial biosynthesis, and maintain mitochondrial homeostasis.

Mitochondrial ATP was measured as an indicator of mitochondrial bioenergetic function (Li et al. 2021). It has been reported that mitochondrial exposure to excessive amount of DIC causes severe ATP reduction, oxidative stress augmentation, and proximal tubular cell death causing kidney damage (Heidari 2019). Possible causes of decreased ATP in kidney injury are as follows: little ATP require reduced effectiveness of ATP synthesis by mitochondrial morphology and dynamics changes, or little ATP production is an adaptation to tolerate cellular stress (Li et al. 2021). In our study, DIC administration showed a significant drop in mitochondrial ATP compared to the control groups. This is compatible with previous studies of Tran et al. (2011) and Patil et al. (2014). ART administration in either prophylactic or therapeutic groups produced significant increase the ATP level supporting the beneficial effect of ART on mitochondrial renal tissue.

Notably, there is mounting evidence to support the crucial role of SIRT3 in the maintenance of mitochondrial homeostasis through modulating mitochondrial dynamics and mitochondrial biogenesis (Yuan et al. 2024). Moreover, SIRT3 deacetylates PGC1-α and mitochondrial complex I, improve mitochondrial biogenesis for kidney injury recovery (Morigi et al. 2018). Thus, supporting the potential impact of ART on kidney injury could be via restoration of mitochondrial homeostasis in SIRT3-dependent manner.

Maintaining cellular homeostasis is achieved through autophagy, a fundamental cyto-protective mechanism. Cellular stress causes it to degrade and recycle damaged cytoplasmic components. The harmful impact of defective autophagy in renal injury and the beneficial benefits of autophagy stimulation on disease progression are both supported by growing data (Gong et al. 2020). The current findings revealed that DIC significantly decreased LC3-II levels revealed by immunostaining, indicating a blockade of autophagic flux, and this was in accordance with the result of El-Maadawy et al. (2022). However, ART administration either in prophylactic or therapeutic groups caused significant increase LC3 II immunostaining, indicating increased autophagy activity. In the same vein, previous results of Feng et al. (2022) who reported that ART increased the expression of LC3II/LC3I, accelerated autophagy by inhibiting the PI3K/AKT/mTOR signaling pathway in diabetic nephropathy animal model. Moreover, SIRT3 promotes autophagy through AMPK/mTOR pathway regulation and LC3 II stimulation, which protect against kidney injury (Xi et al. 2024).

Apoptosis occurs via endogenous pathways mediated by mitochondria as well as exogenous pathways mediated by death receptors. DIC administration caused significant increase of the immunostaining of caspase-3, a pro-apoptotic factor, and this was in accordance with previous results of Huo et al. (2020) and Hashem et al. (2021). ART administration either in prophylactic or therapeutic groups significantly reversed caspase-3 immunostaining; this is in accordance with the result of Zhao et al. (2019). Numerous studies have shown that SIRT3 inhibits caspase-3 activation, which is not surprising considering its function as a downstream mediator and an essential connection between the death receptor and the mitochondrial apoptotic pathways (Li et al. 2017; Kim et al. 2018). Also, it has been reported that SIRT3 inhibits the mitochondrial apoptotic process, preventing kidney damage. Through a mechanism that depends on the mitochondria, excessively acetylated p53 in AKI selectively targets the mitochondria, causing mitochondrial malfunction and death. Nevertheless, it has been demonstrated that SIRT3 effectively deacetylates p53, reducing p53-induced mitochondrial damage (Ouyang et al. 2019).

Finally, structural evaluation of kidney tissue was exhibited in this study by histological and ultra-structural study by EM. DIC administration revealed major changes in H&E histological structure of the kidney as congestion of the glomerular capillaries and many apoptotic cells in the renal tubules; this was the same as the previous results of Abiola et al. (2019), Mostafa et al. (2020), and Nouri et al. (2021). Also PAS stain, the DIC group showed intra-tubular PAS positive casts and thickening of the glomerular and tubular basement membranes this is the same as the result of Fattori et al. (2017). ART administration in either prophylactic or therapeutic groups caused marvelous improvement in kidney tissue by both H&E and PAS stain and this is in agreement of other previous studies (Han et al. 2021).

On EM evaluation, the DIC group showed glomerular basement membrane thickening, which may be caused by large surface area of capillaries of the glomeruli making them more prone to impairment by circulating toxins (Abdel Rahman et al. 2017). Podocyte displayed irregular nucleus and fused foot processes. These alterations are linked to findings of Sayed et al. (2019). ART administration in either prophylactic or therapeutic groups recovered all these changes this is the same as previous results of Wu et al. (2014), Liu et al. (2017), and Han et al. (2021). The potential beneficial effects of ART on structural kidney injury may be related to SIRT3 overexpression inhibiting renal injury (Fan et al. 2017) and its anti-inflammatory, antioxidant, renal autophagy improvement and inhibition of mitogen-activated protein kinase/extracellular signal regulated kinase (MAPK/Erk) pathways (Feng et al. 2022).

## Conclusion

**Fig. 10:**
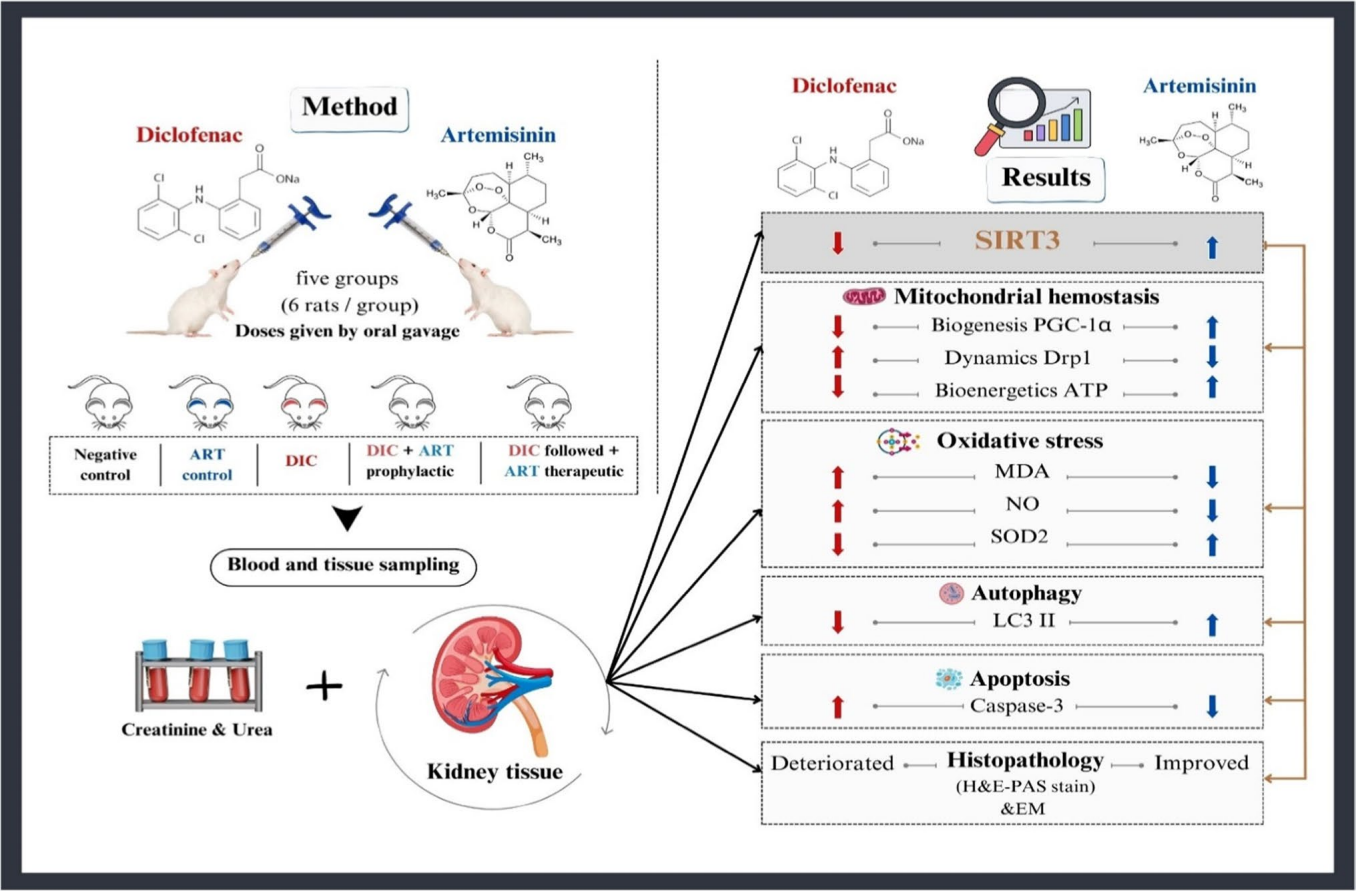
A schematic diagram illustrating the possible mechanisms implicated in the potential role of ART against DIC-induced kidney injury

Hence, we could postulate that the potential effect of ART on mitigating mitochondrial homeostasis, regulation of redox imbalance, restoring autophagy and suppression of apoptosis in DIC-induced renal injury model was due to its positive impact on SIRT3 expression. Furthermore, the current study revealed that prophylactic ART administration showed exhibited significant improvement than therapeutic ART administration suggesting that early management before disease established is better. ART protect against DIC-induced kidney injury, and it is a prospective therapeutic approach in kidney injury (Fig. 10).

## Data Availability

All source data for this work (or generated in this study) are available upon reasonable request.

## Abbreviations

ART: Artemisinin
DIC: Diclofenac
ELISA: Enzyme-linked immunosorbent assay
MDA: Malondialdehyde
SOD: Superoxide dismutase
SIRT3: Sirtuin 3

## References

[CR1] Abdel Rahman MA, Abdel Atty YH, Abdel Rahman MM, Sabry M (2017) Structure changes induced by gibberellic acid in the renal cortex of adult male albino rats. MOJ Anta Physiol 3:21–27

[CR2] Abiola TS, Adebayo OC, Babalola OO (2019) Diclofenac-induced kidney damage in Wistar rats: involvement of antioxidant mechanism. J Biosci Med 7(12):44

[CR3] Abu Hamad R, Berman S, Hachmo Y, Stark M, Hasan F, Doenyas-Barak K, Efratiet S (2017) Response of renal podocytes to excessive hydrostatic pressure: a pathophysiologic cascade in a malignant hypertension model. Kidney Blood Press Res 42:1104–1118 29224013 10.1159/000485774

[CR4] Ahmed T, Archie SR, Faruk A, Chowdhury FA, Al SA, Ahsan CR (2020) Evaluation of the anti-inflammatory activities of diclofenac sodium, prednisolone and atorvastatin in combination with ascorbic acid. AntiInflamm Antiallergy Agents Med Chem 19:291–301 31084596 10.2174/1871523018666190514112048 PMC7499360

[CR5] Alabi QK, Akomolafe RO (2020) Kolaviron diminishes diclofenac-induced liver and kidney toxicity in Wistar rats via suppressing inflammatory events, upregulating antioxidant defenses, and improving hematological indices. Dose Response 18(1):1559325819899256 32165871 10.1177/1559325819899256 PMC7054740

[CR6] Amini N, Badavi M, Goudarzi M (2022) A new combination of naringin and trimetazidine protect kidney Mitochondria dysfunction induced by renal ischemia / reperfusion injury in rat Braz. J Pharm Sci 58:e19870

[CR7] Amini N, Nejaddehbashi F, Badavi M, Bayati V (2024) Zahra Basir4 combined effect of naringin and adipose tissue-derived mesenchymal stem cell on cisplatin nephrotoxicity through Sirtuin1/Nrf-2/HO-1 signaling pathway: a promising nephroprotective candidate. Cell Tissue Res 397:193–204. 10.1007/s00441-024-03902-w 38953985 10.1007/s00441-024-03902-w

[CR8] An Y, Li Q, Sui CH, Wang C (2017) Effects of artemisinin intragastric administration on acute kidney injury induced by cisplatin in mice. Shandong Med J 57(11):36–39. 10.3969/j.issn.1002-266X.2017.11.011

[CR9] Andreas R, Rudiman R, Lukman K, Sulthana BAAS, Purnama A, Putra MRA, Nugraha P, Primastari E (2023) Different clinicopathological characteristics in Indonesian colorectal patients with RAS mutations and LC3 over-expression: a cross-sectional study. Bali Med J 12(2):1774–1780. 10.15562/bmj.v12i2.4519

[CR10] Barzan M, Heydari M, Mirshekari-Jahangiri H, Firouzi H, Dastan M, Najafi M, Khaledi M, Nouri A, Shah-Abadi ME (2023) Carvacrol exerts anti-inflammatory, anti-oxidative stress and hepatoprotective effects against diclofenac-induced liver injury in male rats. Int J Prev Med 14(1):61 37351047 10.4103/ijpvm.ijpvm_178_21 PMC10284240

[CR11] Bozzola JJ, Russel LD (1999) Specimen preparation for transmission electron microscopy. Electron microscopy, 2nd edn. John Pow Company, Canada, pp 16–48

[CR12] Cheng Z, Qi R, Li L, Liu Q, Zhang W, Zhou X, Xu D, Allen TD, Pan S, Liu J (2018) Dihydroartemisinin ameliorates sepsis-induced hyperpermeability of glomerular endothelium via up-regulation of occludin expression. BioMed Pharmacother 99:313–318. 10.1016/j.biopha.2018.01.078 29353206 10.1016/j.biopha.2018.01.078

[CR13] Ding J, Yu HL, Ma WW, Xi YD, Zhao X, Yuan LH, Feng JF, Xiao R (2013) Soy isoflavone attenuates brain mitochondrial oxidative stress induced by beta-amyloid peptides 1–42 injection in lateral cerebral ventricle. J Neurosci Res 91:562–567. 10.1002/jnr.23163 23239252 10.1002/jnr.23163

[CR14] El-Maadawy W, Hassan M, Abdou RM, El-Dine RS, Aboushousha T, El-Tanbouly N et al (2022) 6-Paradol alleviates Diclofenac-induced acute kidney injury via autophagy enhancement-mediated by AMPK/AKT/mTOR and NLRP3 inflammasome pathways. Environ Pharmacol Toxicol 91:103817 10.1016/j.etap.2022.103817 35091105

[CR15] Fan D, Yang Z, Liu FY, Jin YG, Zhang N, Ni J et al (2017) Sesamin protects against cardiac remodeling via Sirt3/ROS pathway. Cell Physiol Biochem 44:2212–2227 29248930 10.1159/000486026

[CR16] Fan H, Le JW, Sun M, Zhu JH (2021) Sirtuin 3 deficiency promotes acute kidney injury induced by sepsis via mitochondrial dysfunction and apoptosis. Iran J Basic Med Sci 24(5):675 34249270 10.22038/ijbms.2021.54905.12312 PMC8244614

[CR17] Farrag EA, Hammad MO, Safwat SM, Hamed S, Hellal D (2023) Artemisinin attenuates type 2 diabetic cardiomyopathy in rats through modulation of AGE-RAGE/HMGB-1 signaling pathway. Sci Rep 13(1):11043 37422477 10.1038/s41598-023-37678-w PMC10329689

[CR18] Fattori V, Borghi SM, Guazelli CFS, Giroldoa AC, Crespigio J, Bussmannc AJC, Coelho-Silvaa L, Ludwig NG, Mazzuco TL, Casagrande R, Verri WA (2017) Vinpocetine reduces diclofenac-induced acute kidney injury through inhibition of oxidative stress, apoptosis, cytokine production, and NF-B activation in mice. Pharmacol Res 120:10–22 28315429 10.1016/j.phrs.2016.12.039

[CR19] Feng H, Wu T, Qi Z, Li H, Liu T, Ma X, Yue R (2022) Protective effect and possible mechanisms of artemisinin and its derivatives for diabetic nephropathy: a systematic review and meta-analysis in animal models. Oxidative Med Cell Longev 2022:5401760 10.1155/2022/5401760 PMC9073547 35528521

[CR20] Funk JA, Schnellmann RG (2012) Persistent disruption of mitochondrial homeostasis after acute kidney injury. Am J Physiol Renal Physiol 302:F853–F864 22160772 10.1152/ajprenal.00035.2011 PMC3340936

[CR21] Galvan DL, Green NH, Danesh FR (2017) Hallmarks of mitochondrial dysfunction in chronic kidney disease. Kidney Int 92:1051–1057 28893420 10.1016/j.kint.2017.05.034 PMC5667560

[CR22] Gong L, Pan Q, Yang N (2020) Autophagy and inflammation regulation in acute kidney injury. Front Physiol 11:576463 33101057 10.3389/fphys.2020.576463 PMC7546328

[CR23] Halling JF, Pilegaard H (2020) PGC-1α-mediated regulation of mitochondrial function and physiological implications. Appl Physiol Nutr Metab 45(9):927–936 32516539 10.1139/apnm-2020-0005

[CR24] Han P, Cai Y, Wang Y, Weng W, Chen Y, Wang M, Zhan H, Yu X, Wang T, Shao M, Sun H (2021) artemether ameliorates kidney injury by restoring redox imbalance and improving mitochondrial function in Adriamycin nephropathy in mice. Sci Rep 11:1266 33446820 10.1038/s41598-020-80298-x PMC7809108

[CR25] Harirforoosh S, West KO, Murrell DE, Denham JW, Panus PC, Hanley GA (2016) Examination of the pharmacodynamics and pharmacokinetics of a diclofenac poly(lactic-co-glycolic) acid nanoparticle formulation in the rat. Eur Rev Med Pharmacol Sci 20(23):5021–5031 27981527

[CR26] Hashem KS, Abdelazem AZ, Mohammed MA, Nagi MA, Aboulhoda BE, Mohammed ET et al (2021) Thymoquinone alleviates mitochondrial viability and apoptosis in diclofenac-induced acute kidney injury (AKI) via regulating Mfn2 and miR-34a mRNA expressions. Environ Sci Pollut Res 28:10100–10113 10.1007/s11356-020-11313-x 33165700

[CR27] Heidari R (2019) The footprints of mitochondrial impairment and cellular energy crisis in the pathogenesis of xenobiotics-induced nephrotoxicity, serum electrolytes imbalance, and Fanconi’s syndrome: a comprehensive review. Toxicology 423:1–31. 10.1016/j.tox.2019.05.002 31095988 10.1016/j.tox.2019.05.002

[CR28] Huo X, Meng Q, Wang C, Wu J, Wang C, Zhu Y, Ma X, Sun H, Liu K (2020) Protective effect of cilastatin against diclofenac-induced nephrotoxicity through interaction with diclofenac acyl glucuronide via organic anion transporters. Br J Pharmacol 177:1933–1948 32000294 10.1111/bph.14957 PMC7161545

[CR29] Juszczak F, Arnould T, Declèves AE (2024) The role of mitochondrial sirtuins (sirt3, sirt4 and sirt5) in renal cell metabolism: implication for kidney diseases. Int J Mol Sci 25(13):6936 39000044 10.3390/ijms25136936 PMC11241570

[CR30] Kim D, Park W, Lee S, Kim W, Park SK, Kang KP (2018) Absence of Sirt3 aggravates cisplatin nephrotoxicity via enhanced renal tubular apoptosis and inflammation. Mol Med Rep 18(4):3665–3672 30106119 10.3892/mmr.2018.9350 PMC6131565

[CR31] Kuo J (2007) Methods in molecular biology. Electron microscopy methods and protocols, 2nd edn. Humana Press, New Jersey, p 67

[CR32] Li Y, Ye Z, Lai W, Rao J, Huang W, Zhang X, Lou T (2017) Activation of sirtuin 3 by silybin attenuates mitochondrial dysfunction in cisplatin-induced acute kidney injury. Front Pharmacol 8:178 28424621 10.3389/fphar.2017.00178 PMC5380914

[CR33] Li L, Chen X, Dong F, Liu Q, Zhang C, Xu D, Allen TD, Liu J (2018) Dihydroartemisinin up-regulates VE-cadherin expression in human renal glomerular endothelial cells. J Cell Mol Med 22(3):2028–2032. 10.1111/jcmm.13448 29193726 10.1111/jcmm.13448 PMC5824371

[CR34] Li Y, Hepokoski M, Gu W, Simonson T, Singh P (2021) Targeting mitochondria and metabolism in acute kidney injury. J Clin Med 10:3991 34501442 10.3390/jcm10173991 PMC8432487

[CR35] Liu LZ, Zhang MX, Wang S (2017) Effects of artemisinin on excretion of nephrin, podocin mRNA and urinary podocyte cells in Heymann nephritis rats. Chin Traditional Patent Med 39(10):2176–2178. 10.3969/j.issn.1001-1528.2017.10.041

[CR36] Liu X, Lu J, Liao Y, Liu S, Chen Y, He R, Men L, Lu C, Chen Z, Li S, Xiong G, Yang S (2019) Dihydroartemisinin attenuates lipopolysaccharide-induced acute kidney injury by inhibiting inflammation and oxidative stress. BioMed Pharmacother 117:109070. 10.1016/j.biopha.2019.109070 31176164 10.1016/j.biopha.2019.109070

[CR37] Livak KJ, Schmittgen TD (2001) Analysis of relative gene expression data using realtime quantitative PCR and the 2 (-Delta Delta C (T)) Method. Methods 25:402–408. 10.1006/meth.2001.1262 11846609 10.1006/meth.2001.1262

[CR38] Locatelli M, Zoja C, Zanchi C, Corna D, Villa S, Bolognini S, Novelli R et al (2020) Manipulating Sirtuin 3 pathway ameliorates renal damage in experimental diabetes. Sci Rep 10:8418 32439965 10.1038/s41598-020-65423-0 PMC7242337

[CR39] Lowry OH, Rosenbrough NJ, Farr AL, Randall RJ (1951) Protein measurement with the Folin phenol reagent. J Biol Chem 193:265–275 14907713

[CR40] Luisi G (2023) Antimalarial endoperoxides: from natural sesquiterpene drugs to a rising generation of synthetic congeners. Terpenes 2:158–227

[CR41] Lyamzaev KG, Zinovkin RA, Chernyak BV (2022) Extrusion of mitochondria: Garbage clearance or cell–cell communication signals? J Cell Physiol 237(5):2345–2356 35253232 10.1002/jcp.30711

[CR42] McMahon SB, Dargan P, Lanas A, Wiffen P (2021) The burden of musculoskeletal pain and the role of topical non-steroidal anti-inflammatory drugs (NSAIDs) in its treatment. Ten underpinning statements from a global pain faculty. Curr Med Res Opin 37(2):287–292 33155849 10.1080/03007995.2020.1847718

[CR43] Meng Y, Ma N, Lyu H, Wong YK, Zhang X, Zhu Y, Gao P, Sun P, Song Y, Lin L, Wang J (2021) Recent pharmacological advances in the repurposing of artemisinin drugs. Med Res Rev 41(6):3156–3181 34148245 10.1002/med.21837

[CR44] Miao C, Zhu X, Wei X, Long M, Jiang L, Li C, Jin D, Du Y (2022) Pro-and anti-fibrotic effects of vascular endothelial growth factor in chronic kidney diseases. Ren Fail 44(1):881–892 35618410 10.1080/0886022X.2022.2079528 PMC9154791

[CR45] Morigi M, Perico L, Benigni A (2018) Sirtuins in renal health and disease. J Am Soc Nephrol 29:1799–1809. 10.1681/ASN.2017111218 29712732 10.1681/ASN.2017111218 PMC6050939

[CR46] Mostafa RE, El-Marasy SA, Abdel Jaleel GA, Bakeer RM (2020) Protective effect of royal jelly against diclofenac-induced hepato-renal damage and gastrointestinal ulcerations in rats. Heliyon 6(2):E03330 32025584 10.1016/j.heliyon.2020.e03330 PMC6997571

[CR47] Nouri A, Heidarian E (2019a) Ameliorative effects of N-acetyl cysteine on diclofenac induced renal injury in male rats based on serum biochemical parameters, oxidative biomarkers, and histopathological study. J Food Biochem 43:e12950 31368551 10.1111/jfbc.12950

[CR48] Nouri A, Heidarian E (2019b) Nephro-protective effect of silymarin against diclofenac induced renal damage and oxidative stress in male rats. J Herbmed Pharmacol 8(2):146–152

[CR49] Nouri A, Izak-Shirian F, Fanaei V, Dastan M, Abolfathi M, Moradi A, Khaledi M, Mirshekari-Jahangiri H (2021) Carvacrol exerts nephroprotective effect in rat model of diclofenac-induced renal injury through regulation of oxidative stress and suppression of inflammatory response. Heliyon 7:e08358 34816045 10.1016/j.heliyon.2021.e08358 PMC8591494

[CR50] Ogbe RJ, Luka CD, Adoga GI (2019) Effect of aqueous ethanol extract of Dialium guineense leaf on diclofenac-induced oxidative stress and hepatorenal injuries in Wistar rats. Comp Clin Pathol 28:241–248

[CR51] Ok Atılgan A, Tepeoğlu M, Yılmaz Akçay E, Hasanaliyeva L, Kılıç D, Özdemir H (2023) The expression of caspase-3 and GRIM-19 in non-mucinous lung adenocarcinoma and their clinicopathologic significance. Duzce Med J 25:158–166. 10.18678/dtfd.1294988

[CR52] Okamura DM, Pennathur S (2015) Te balance of powers: redox regulation of fbrogenic pathways in kidney injury. Redox Biol 6:495–504 26448394 10.1016/j.redox.2015.09.039 PMC4600846

[CR53] Ouyang J, Zeng Z, Fang H, Li F, Zhang X, Tan W (2019) SIRT3 inactivation promotes acute kidney injury through elevated acetylation of SOD2 and p53. J Surg Res 233:221–230 30502252 10.1016/j.jss.2018.07.019

[CR54] Patil NK, Parajuli N, MacMillan-Crow LA, Mayeux PR (2014) Inactivation of renal mitochondrial respiratory complexes and manganese superoxide dismutase during sepsis: mitochondria-targeted antioxidant mitigates injury. Am J Physiol Renal Physiol 306:F734–F743 24500690 10.1152/ajprenal.00643.2013 PMC3962604

[CR55] Perry HM, Huang L, Wilson RJ, Amandeepe B, Hiromi S, Zhen Y, Diane LR, David FK, Mark DO (2018) Dynamin-related protein 1 deficiency promotes recovery from AKI. J Am Soc Nephrol. 29(1):194–206. 10.1681/ASN.2017060659 29084809 10.1681/ASN.2017060659 PMC5748924

[CR56] Qin YR, Ma CQ, Jiang JH, Wang DP, Zhang QQ, Liu MR, Liu Y (2022) Artesunate restores mitochondrial fusion-fission dynamics and alleviates neuronal injury in Alzheimer’s disease models. J Neurochem 162(3):290–304 35598091 10.1111/jnc.15620

[CR57] Ruiz S, Pergola PE, Zager RA, Vaziri ND (2013) Targeting the transcription factor Nrf2 to ameliorate oxidative stress and inflammation in chronic kidney disease. Kidney Int 83(6):1029–1041. 10.1038/ki.2012.439 23325084 10.1038/ki.2012.439 PMC3633725

[CR58] Sayed H, Hoda A, Heba M, Martha A (2019) Effects of exposure to gibberellic acid during pregnancy and lactation on the postnatal development of the renal cortex in the albino rat. JCMRP. 4(2):121–130

[CR59] Scholz H, Boivin FJ, Schmidt-Ott KM, Bachmann S, Eckardt KU, Scholl UI, Persson PB (2021) Kidney physiology and susceptibility to acute kidney injury: implications for renoprotection. Nat Rev Nephrol 17(5):335–349 33547418 10.1038/s41581-021-00394-7

[CR60] Sivaraj R, Umarani S (2018) Diclofenac-induced biochemical changes in nephrotoxicity among male Albino rats. Int J Basic ClinPharmacol 7:640–643

[CR61] Szeto HH, Liu S, Soong Y, Seshan SV, Cohen-Gould L, Manichev V, Feldman LC, Gustafsson T (2017) Mitochondria protection after acute ischemia prevents prolonged upregulation of IL-1beta and IL-18 and arrests CKD. J Am Soc Nephrol 28:1437–1449 27881606 10.1681/ASN.2016070761 PMC5407729

[CR62] Tran M, Tam D, Bardia A, Bhasin M, Rowe GC, Kher A, Zsengeller ZK (2011) PGC-1alpha promotes recovery after acute kidney injury during systemic inflammation in mice. J Clin Investig 121:4003–4014 21881206 10.1172/JCI58662 PMC3195479

[CR63] Varrier M, Fisher R, Ostermann M (2015) Acute kidney injury-an update. EMJ Nephrol 3(1):75–82

[CR64] Verhoeven F, Totoson P, Marie C, Prigent-Tessier A, Wendling D, Tournier- Nappey M, Pratiet C, Demougeot C (2017) Diclofenac but not celecoxib improves endothelial function in rheumatoid arthritis: a study in adjuvant-induced arthritis. Atherosclerosis 266:136–144 29024866 10.1016/j.atherosclerosis.2017.09.033

[CR65] Wai T, Langer T (2016) Mitochondrial dynamics and metabolic regulation. Trends Endocrinol Metab 27:105–117 26754340 10.1016/j.tem.2015.12.001

[CR66] Wang Y, Han P, Wang M, Weng W, Zhan H, Yu X, Yuan C, Shao M, Sun H (2019) Artemether improves type 1 diabetic kidney disease by regulating mitochondrial function. Am J Transl Res 11(6):3879–3889 31312396 PMC6614617

[CR67] Whitaker RM, Corum D, Beeson CC, Schnellmann RG (2016) Mitochondrial biogenesis as a pharmacological target: a new approach to acute and chronic diseases. Annu Rev Pharmacol Toxicol 56:229–249 26566156 10.1146/annurev-pharmtox-010715-103155

[CR68] Wu X, An P, Ye B, Shi X, Dang H, Fu R et al (2014) Artemisinin ameliorated proteinuria in rats with Adriamycin-induced nephropathy through regulating nephrin and podocin expressions. J Tradit Chin Med 34(1):63–68. 10.1016/S0254-6272(14)60056-X 25102693 10.1016/s0254-6272(14)60056-x

[CR69] Xi S, Chen W, Ke Y (2024) Advances in SIRT3 involvement in regulating autophagy-related mechanisms. Cell Div 19:20. 10.1186/s13008-024-00124-y 38867228 10.1186/s13008-024-00124-y PMC11170824

[CR70] Xia M, Liu D, Liu Y, Liu H (2020) the therapeutic effect of artemisinin and its derivatives in kidney disease. Front Pharmacol 11:380 32296335 10.3389/fphar.2020.00380 PMC7136752

[CR71] Yuan J, Zhao J, Qin Y, Zhang Y, Wang A, Ma R, Sun S (2024) The protective mechanism of SIRT3 and potential therapy in acute kidney injury. QJM Int J Med 117(4):247–255 10.1093/qjmed/hcad152 37354530

[CR72] Zhang Q, Liu X, Zhang J, Yang J, Bu P (2018) Sirtuin 3 deficiency aggravates contrast-induced acute kidney injury. J Transl Med 16(1):1–12 30445987 10.1186/s12967-018-1690-5 PMC6240230

[CR73] Zhang H, Qi S, Song Y, Ling C (2020) Artemisinin attenuates early renal damage on diabetic nephropathy rats through suppressing TGF-β1 regulator and activating the Nrf2 signaling pathway. Life Sci 256:117966 32535079 10.1016/j.lfs.2020.117966

[CR74] Zhang S, Feng R, Yuan F, Luo Q, Chen X, Li N et al (2022) the therapeutic effects of dihydroartemisinin on cisplatin-resistant gastric cancer cells. Curr Pharm Biotechnol 23(2):276–286. 10.2174/1389201022666210217114825 33596797 10.2174/1389201022666210217114825

[CR75] Zhao W, Zhang L, Chen R, Lu H, Sui M, Zhu Y, Zeng L (2018) SIRT3 protects against acute kidney injury via AMPK/mTOR-regulated autophagy. Front Physiol 9:1526 30487750 10.3389/fphys.2018.01526 PMC6246697

[CR76] Zhao X, Fang J, Li S, Gaur U, Xing X, Wang H et al (2019) Artemisinin attenuated hydrogen peroxide (H2O2)-induced oxidative injury in SH-SY5Y and hippocampal neurons via the activation of AMPK pathway. Int J Mol Sci 20(11):2680. 10.3390/ijms20112680 31151322 10.3390/ijms20112680 PMC6600327

